# Estimation and prediction for a mechanistic model of measles transmission using particle filtering and maximum likelihood estimation

**DOI:** 10.1002/sim.8290

**Published:** 2019-07-09

**Authors:** Kirsten E. Eilertson, John Fricks, Matthew J. Ferrari

**Affiliations:** ^1^ Statistics Pennsylvania State University State College PA; ^2^ School of Mathematical and Statistical Sciences Arizona State University Tempe AZ; ^3^ Biology Pennsylvania State University State College PA

**Keywords:** measles, particle filter, stochastic model, time series, under‐reporting

## Abstract

Disease incidence reported directly within health systems frequently reflects a partial observation relative to the true incidence in the population. State‐space models present a general framework for inferring both the dynamics of infectious disease processes and the unobserved burden of disease in the population. Here, we present a state‐space model of measles transmission and vaccine‐based interventions at the country‐level and a particle filter‐based estimation procedure. Our dynamic transmission model builds on previous work by incorporating population age‐structure to allow explicit representation of age‐targeted vaccine interventions. We illustrate the performance of estimators of model parameters and predictions of unobserved states on simulated data from two dynamic models: one on the annual time‐scale of observations and one on the biweekly time‐scale of the epidemiological dynamics. We show that our model results in approximately unbiased estimates of unobserved burden and the underreporting rate. We further illustrate the performance of the fitted model for prediction of future disease burden in the next one to 15 years.

## 1 INTRODUCTION

Quantifying the burden of disease is critical to evaluate the performance of public health interventions, such as vaccination, and to inform decisions about the allocation of public health resources. However, many common diseases are difficult to quantify directly as the majority of infections are not reported to health surveillance systems. This is particularly the case in low‐income country settings, which often account for the highest burden of vaccine‐preventable childhood infections. Thus, the burden of disease must be estimated from available information.^1^ Measles vaccination has been called a “best‐buy” in public health^1^, ^2^ and all 5 WHO health regions have goals for the eradication of measles by 2020. Prior to the introduction of measles vaccination, nearly all humans could expect to be infected in their lifetime^3^; the introduction of a stable and relatively inexpensive vaccine has been credited with averting 20.4 million deaths over the period 2000 to 2016 alone.^4^ However, the increase of vaccine coverage rates has stagnated.^4^ Thus, to evaluate progress toward elimination goals, it is crucial to have estimates of the burden of measles infection. Measles cases have been routinely reported by WHO member countries since 1980, though some countries began reporting later; however, these cases, which have been reported to the health system, are generally assumed to be a significant underestimate of the true number of cases occurring in the community. Simons et al^1^ estimated that underreporting varied greatly by country but was frequently below 10%. Thus, the primary goal of burden estimation is to provide quantitative estimates of the unobservable number of infections that are not reported to the health system.

Chen et al^5^ presented a state‐space model, also known as a hidden Markov model, to predict the unobserved burden of measles disease from annual time series of reported cases in each country. State space models are characterized by two interrelated parts: a state model, which is typically a Markov process, and an observation model, which is a random variable that depends on the state at the current time. Typically, the observations are independent when conditioning on the states. These assumptions about the structure of the model will allow us to carry out statistical inference on the parameters and predict the unobserved states based only on the conditional probability densities *p*(*x*_*t*_|*x*_*t*−1_) and *p*(*y*_*t*_|*x*_*t*_). In the model presented by Chen et al,^5^ the state *X*_*t*_ represents the unobservable burden of measles infection with *f* (*X*_*t*−1_) describing the conditional mean of *X*_*t*_ and, thus, the dynamic progression of disease burden as a function of transmission and demographic rates. The observation, *Y*_*t*_, represents the reported number of cases, and *g*(*X*_*t*_) describes the conditional mean of *Y*_*t*_, interpreted as the country‐specific fraction of cases that are reported to the health system. Chen et al fit this model using maximum likelihood estimation, with an extended Kalman filter (EKF) to calculate the likelihood.

Though the EKF provides computational and structural simplicity, the model presented by Chen et al^5^ made several simplifying assumptions that are imperfect for the epidemiology of measles transmission and representation of measles vaccination. In particular, the EKF method as presented in that work assumes that the stochastic terms in both the process and observation models are conditionally Gaussian with constant variance through time. As with many counting processes, it would be reasonable to assume that the variation in both true and observed number of measles cases scaled with the level of the process; in other words, the higher the population, the more variation one would expect in the change of the population.

Vaccination strategies for measles involve multiple doses delivered through either a routine or supplemental immunization system. In the former, children are encouraged to visit clinics at specific ages to receive a first and, possibly, second dose of measles containing vaccine. In the supplemental system, large short‐term campaigns are conducted, which seek to vaccinate all, or most, children in specified target ages. These campaigns are designed to provide a second (or third) dose opportunity for children vaccinated through the routine system and a first dose opportunity to those children missed by the routine system. The targeted ages vary from country to country and campaign to campaign based on logistical capacity and expected risk. While the coverage of supplemental campaigns describes the number of children vaccinated, the impact of these campaigns on increased rates of immunization depends on the rate of immunization through the routine system (a child successfully immunized through the routine system receives no additional benefit through a supplemental dose) and rate of natural infection (a child that has previously been infected with measles is immune and also receives no benefit from a supplemental dose).^6^ The model of Chen et al^5^ did not explicitly represent the age distribution of the susceptible population, thus the impact of these campaigns on increased immunization was represented implicitly.

Here, we present a state‐space model that addresses these two limitations of the previous work by Chen et al.^5^ Specifically, we represent the infection and reporting processes with binomial models, which both constrain the states to a natural scale (infections must be a subset of the susceptible population and observed cases must be a subset of total cases) and scales the variance of the stochastic term with the state of the system. The higher the number in a population, the more variation one expects when moving from one time point to the next. Furthermore, we represent age‐classes within states, which allows for explicit representation of age‐specific vaccination strategies. First, we present the basic model for progression of measles through time and then discuss our approach to fitting the model using maximum likelihood estimation, with a particle filter to predict the unobserved burden and to calculate the likelihood. We then illustrate the performance of the model to estimate measles incidence from simulated data and to predict future measles burden from the fitted model.

## 2 MODEL

**Figure 1:**
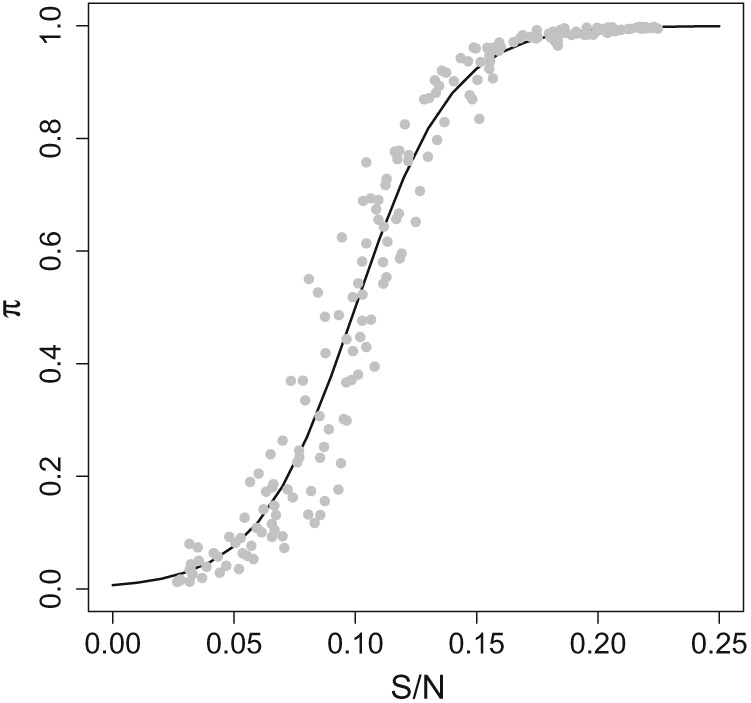
An example curve that illustrates the attack rate function for the annual model. The points illustrate the attack rate variation, $\sigma_{e}^{2}$

The natural history of measles transmission is often represented using the Susceptible‐Infected‐Recovered (SIR) modeling framework.^7^ Individuals are assumed to belong to one of three classes: susceptible (S), infected (I), and recovered (R, who are immune to subsequent infection).^7^ Conventional representations of SIR‐style models assume that the individuals within the population are “well‐mixed”. These include both deterministic forms represented as ordinary differential equations^7^ or stochastic forms such as the time series SIR (TSIR) model.^8^ Such interactions among individuals can be approximated by mass action kinetics.^9^ Large aggregations, such as countries, are unlikely to be well represented by the mass action assumption, though the interactions among many subunits (eg, municipalities within a country) have been well described as a metapopulation.^10^ Explicit representation of any specific metapopulation requires knowledge of the rates of interaction (eg, migration, commuting) among subunits.^11^ As such high resolution information is rarely consistently available at national scale, we here present a phenomenological model for dynamics at the national scale to support national estimation of the burden of measles disease. The form of this model, presented later and illustrated in Figure 1, has been chosen to reflect the behavior captured by conventional SIR‐type models and metapopulation models, though simple and flexible enough to encompass the variety of dynamics observed in diverse countries.

We group the population into *A* age groups. In our notation, superscript *a* indicates an age‐group specific calculation. For example, $S_{t}^{a}$ is the number of susceptible individuals in age group *a* in year *t*, and *S*_*t*_ would be the total number susceptible in year *t*. The number of children susceptible in age class *a* in year *t* can be modeled as a function of the number susceptible from year *t*−1, the number of births that year, *B*, the impact of vaccination, *V*(), which moves children of age *a* from the susceptible to the recovered class, and infections from the previous year *I*, ie,

(1) $$S_{t}^{a}=S_{t-1}^{a-1}+V^{a}\left(B_{t},S_{t-1}^{\;a-1}\right)-I_{t-1}^{a-1}.$$

Our model specifies the impact of vaccines on the number of susceptible individuals with the function $V^{a}(B_{t},S_{t-1}^{a})$. This function is assumed to be known. It reflects the routine vaccination that occurs each year and is administered to infants, *B*_*t*_, as part of a routine vaccination schedule. *V* ^*a*^() also includes sporadic age‐targeted supplemental vaccination activities, which occur occasionally and target all children within specific age classes, $S_{t}^{a}$. The function $V^{a}(B_{t},S_{t-1}^{\;a-1})$ specifies the fraction of each age class, *a*, that is immunized through vaccination in a given year, *t*. The fraction immunized is itself a function of the vaccination coverage (the proportion of children who receive a vaccine) and the vaccine effectiveness (the proportion of individuals receiving vaccine that develop protective immunity). The vaccine effectiveness is itself age‐specific because of the potential interaction with maternally derived antibodies in infants.^12^ A summary of model terms can be found in Table 1.

**Table 1:** Description of model terms

**Term**	**Description**
*a*	Index of Age group
*t*	Index of Year
$S_{t}^{a}$	Unobserved number of Susceptible at time t, in age group a
$I_{t}^{a}$	Unobserved number of Infected at time t, in age group a
*C*_*t*_	Observed number of Cases reported in year t
*V* ^*a*^()	Specifies the fraction of each age class immunized through vaccination
*e*_*t*_	Effect of unspecified influences on attack rate
*π*_*t*_	Attack rate in year t
*p*_*r*_	Probability of reporting a case
*β*_0_	Parameter in attack rate function
*β*_1_	Parameter in attack rate function
$\sigma_{e}^{2}$	Attack rate variation

We assume that the fraction of susceptible individuals that become infected each year is given by an attack rate that is an increasing function of the fraction of the population that is susceptible, *S*_*t*_/*N*_*t*_. Thus, the number of measles cases in age class *a* in year *t* modeled as a binomial draw from the susceptible population $S_{t}^{a}$, ie,

(2) $$\begin{array}{ll} I_{t}^{a} & ∼Bin\left(S_{t}^{a},\pi_{t}\right)\\ \pi_{t} & ={\text{logit}}^{-1}\left(\beta_{0}+\beta_{1}\frac{S_{t}}{N_{t}}+e_{t}\right), \end{array}$$

where $e_{t}∼N(0,\sigma_{e}^{2}),t=1,2,\text{…},T$ are independent random variables and the $I_{t}^{a}$ depend on the past only through $S_{t}^{a}$ and *π*_*t*_. This logistic form reflects the indirect protection of herd immunity as the fraction of the population that is immune increases. The standard SIR‐model formulation has a critical threshold immunity, 1−1/*R*_0_, where *R*_0_ is the basic reproductive ratio, above which endemic transmission cannot persist. In a metapopulation, however, it is possible for endemic transmission to persist through a process of repeated local extinction and reintroduction.^10^ Furthermore, even in countries that have achieved the documented elimination of endemic transmission, low numbers of cases are possible through importation from endemic countries.^13^ Thus, rather than attempting a functional form that can account for explicit dynamics of within and between country migration, we rely on this phenomenological form, which incorporates indirect protection via the logit model and allows other unspecified mechanisms to be captured in a noise term, *e*_*t*_.

The surveillance data provide the number of reported cases, *C*_*t*_, summed over all age classes, which is assumed to be a subset of the number of actual cases, many of which are not seen by the health system,

(3) $$C_{t}∼Bin(I_{t},p_{r}),$$

where $I_{t}=\sum_{a}I_{a,t}$ and the *C*_*t*_ depends on the past only through *I*_*t*_. We assume that cases are reported with probability *p*_*r*_, where *p*_*r*_ is specific to each country and generally assumed to be constant over time.

## 3 ESTIMATION AND PREDICTION

For prediction of unobserved states and estimation of parameters, we use a particle filter to predict the unobserved states of the system, a calculation that also gives a Monte Carlo approximation of the likelihood function, which we can then maximize. We will redefine some of our notation to conform to more traditional state space notation. We define *X*_*t*_ to be the vector of the unobserved values for the number of susceptible and the number of infected at time *t*; *X*_*t*_=(*S*_*t*_,*I*_*t*_). We will refer to our observed data, *C*_*t*_, as *Y*_*t*_.

### 3.1 Estimation of parameters

We employ a particle filter^14^ to approximate the likelihood of the model specified in Equations 1, 2, and 3, for a given set of parameter values for *β*_0_, *β*_1_, *p*_*r*_, *σ*_*e*_. A filter in this context allows us to calculate the conditional density of the state of a system, *X*_*t*_, at time *t* given the observed part of the system up to time *t* ≤ *T*, *Y*_1:*t*_={*Y*_1_,…,*Y*_*t*_}. This is known as the filtering density, *p*(*x*_*t*_|*Y*_1:*t*_), and we approximate this by sequential importance sampling. The procedure is as follows.

1. Initialize by sampling *R* particles (proposed values) from a initial probability distribution, *p*(*x*_0_), for our unobserved state, *X*_0_. We use $X_{0}^{j}$ to indicate the *j*th particle.
2. For each *j* from one to *R*, draw $X_{t}^{j}$ from $p(x_{t}|X_{t-1}^{j})$ In this step, each particle, $X_{t-1}^{j}$, is advanced one time step according to Equation 1 and Equation 2.
3. Evaluate weights $w_{j}=p(Y_{t}|X_{t}^{j})$.The weight for each of the particles is calculated using the conditional density from our observation model in Equation 3.
4. Resample *R* observations with replacement from $X_{t}^{1},\text{…},X_{t}^{R}$ according to weights *w*_*j*_. Increase *t* by one and return to step 2, unless *t*=*T* in which case terminate the algorithm.

The resampled particles from step 4 approximate the marginal density *p*(*x*_*t*_|*Y*_1:*t*_) (the filtering density). For the initial distribution of *X*_0_, we assume the a fraction of the total population size is susceptible at time t= 0, and draw *S*_0_ from a uniform distribution between 0.1% and 10% of the total population size; this reflects the steady‐state proportion susceptible expected in the absence of vaccination if *R*_0_ ≥ 10. We also assume that, at time zero, the distribution of susceptible individuals across age groups is geometrically distributed, with parameter *q*=.25. This gives a mean age of susceptibles of 4 years in the initial year. Although this is unknown for any country, it is consistent with the mean age of infection for endemic measles^7^, ^15^ and we assume that the observed time series is sufficiently long (>20 years) that the choice of initial conditions will have trivial impact as per the works of Simons et al^1^ and Chen et al.^5^

We obtain maximum likelihood estimates of the parameters *β*_0_, *β*_1_, and *p*_*r*_ via numerical optimization. Let $w_{t}^{j}$ represent the weights calculated in step 3 corresponding to year *t*, particle *j*. The log‐likelihood is proportional to $\sum_{t=1}^{T}\log(\frac{1}{R}\sum_{j=1}^{R}w_{t}^{j})$. Thus, the filter provides for the evaluation of the likelihood function. We identify the optimal combination of *β*_0_,*β*_1_, and *p*_*r*_ by evaluation of the likelihood function on a grid of 2000 parameter value combinations. We then conduct a second grid search on a reduced range of the parameter space with another 2000 parameter combinations. This reduced range corresponds to the parameter space that containts the highest 5% of likelihood values in the initial search.

To estimate the uncertainty in these parameter value estimates, we use parametric bootstrapping, with 100 bootstrapped data sets. To perform the parametric bootstrap, we use the maximum likelihood parameter estimates to generate 100 simulated time series from Equations 1, 2, and 3. We then use the methods described earlier to generate maximum likelihood estimates of *β*_0_, *β*_1_, *p*_*r*_ from the simulated time series. The point estimate for each parameter is then the maximum likelihood estimate from the original data and a 95% confidence interval is the 2.5th and 97.5th percentile of the bootstrapped estimates.

**Figure 2:**
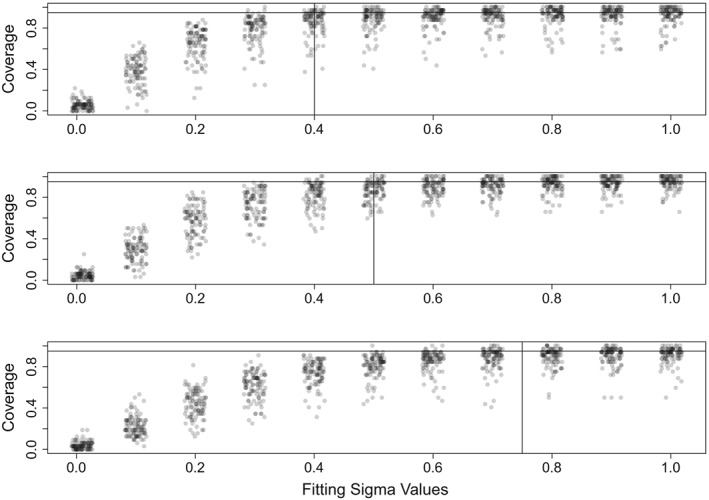
Coverage of nominal 95% intervals for the states generated from the filtering density as a function of the level of attack rate variation, σ_e_, used in the simulation. Vertical black lines indicate the level of σ_e_ used to simulate the observed data

Estimation of $\sigma_{e}^{2}$, which indicates the variability in the attack rate due to unspecified sources, is done separately from the other parameters. We would expect a 95% prediction interval from the one step ahead predictor generated by the true parameter values to cover the unobserved states, *I*, approximately 95% of the time. Thus, we choose $\hat{\sigma_{e}^{2}}$ to be the value that produces 95% coverage of $Î=C/{\hat{p}}_{r}$ from the one step ahead predictor associated with our likelihood parameter estimates, ${\hat{\beta }}_{0},{\hat{\beta }}_{1}$, and ${\hat{p}}_{r}$. As illustrated in Figure 2, simulations indicate that coverage saturates near the nominal coverage in the vicinity of the value of $\sigma_{e}^{2}$ used to generate the simulations. This estimate of $\sigma_{e}^{2}$ will be used for forward projections.

### 3.2 Prediction of states

Predictions of the past measles burden, *I*, are calculated based on the smoothing density: the marginal distribution of the unobserved states for a particular year given all the observed data *p*(*x*_*t*_|*Y*_1:*T*_). We approximate the smoothing distribution using a particle filter procedure as described below.

1. Initialize by sampling *R* particles (proposed values) from an initial probability distribution of our unobserved states, *X*_0_, as described earlier for estimation of parameters.
2. Draw *R* particles from the density $p(x_{t}|X_{t-1}^{j})$ as described earlier for estimation of parameters.
3. Repeat step 2 for each year *t*=2,…,*T*. Each of the *R* particles will now be a matrix of dimension 2 by T, containing that particle's realized value of susceptible and infected individuals for each year. We refer to these matrices as the particle paths.
4. Evaluate $p(Y_{1:T}|X_{1:T}^{j})$ to calculate weight *w*_*j*_ associated with each path. The weight for each of the particle paths is calculated using the likelihood corresponding to our observation model in Equation 3.
5. Resample *R* observations from $X_{1:T}^{1},\text{…},X_{1:T}^{R}$ according to weights *w*_*j*_. In this final step, we are resampling the entire particle path.

The distribution of the resampled observations from step 5 is an approximation to the joint distribution of *X*_1:*T*_|*Y*_1:*T*_ for a given set of parameter values. The distribution of *X*_*t*_|*Y*_1:*T*_ is then also approximated and is known as the smoothing distribution and is the marginal distribution of the state at a particular time step, *X*_*t*_ given all the data *Y*_1:*T*_. A conditional distribution of the unobserved states, which includes the uncertainty in parameter estimates, is calculated by aggregating the smoothing distributions resulting from runs of the particle filter under all 100 of the parametric bootstrapped parameter values (described earlier). Prediction intervals for the unobserved *I*_*t*_ are the central 95% of the bootstrapped smoothing distribution.

### 3.3 Prediction of future states

Prediction of future measles burden is calculated from the prediction distributions: the distribution of the future states (*S*_*T*+*n*_,*I*_*T*+*n*_), n years after the current year of data conditioned on the observations up to time *T*. Importantly, we can vary the coverage and availability of routine measles vaccination programs (MCV1 and MCV2), and supplemental immunization activities (SIAs), in the years to come by adjusting the function *V*. In this way, we can assess the impact of these programs on the measles burden in near term.

## 4 EVALUATION OF METHOD VIA SIMULATION

To assess our ability to estimate parameters and predict past and future infection levels, we use simulated data. The data is generated from two modeling paradigms. The first paradigm described later, the annual model, is the same model we described in Section 2. Using data generated from the annual model allows us to check our model's ability to recover the true parameter values. The second paradigm we use is the more classical SIR model with a two‐week time step, but aggregated to yearly counts.

### 4.1 Annual model

**Figure 3:**
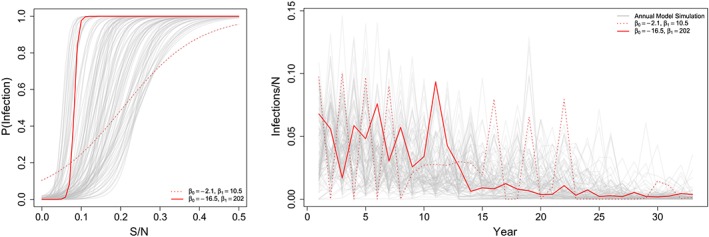
Left: The range of phenomenological annual attack rate functions used in the simulation experiment. Each line represents a sampled parameter set of β_0_ and β_1_ used to generate a data set. Red lines indicate two examples in the range of possible variation. Right: Time series of unobserved incidence cases over time from each of our 100 parameter combinations. The trends in red correspond to two highlighted attack rate curves highlighted in the figure on the left [Colour figure can be viewed at wileyonlinelibrary.com]

To generate candidate parameter values, we fit the annual model to reported case and vaccination data between the 1980 and 2014 from 193 countries. These data are as described in the work of Simons et al.^1^ We do not present any specific analysis of these fits to reported data as our goal here was simply to generate parameters in a plausible range that could replicate time series of reported cases consistent with real‐world observation. We used the resulting distributions of parameter estimates from the work of Simons et al^1^ to generate simulated time series as follows. We sampled 100 pairs of values for *β*_0_ and *β*_1_, and separately assigned values of *p*_*r*_ from a likely range, ie, 0.01, 0.02, 0.03,0.04, and 0.05. The range of attack‐rate curves explored is illustrated in Figure 3.

Each of the 193 countries has reported coverage for both routine measles vaccination programs (MCV1 and MCV2) and SIAs. We sampled 100 of these programs jointly. This information is used to build the function $V^{a}(B_{t},S_{t-1}^{a})$. The first routine dose of measles vaccine was assumed to be administered at 1 year of age, and we assume 85% efficacy. The second routine dose of measles vaccination was assumed to be administered to children at 2 years of age with 95% efficacy. The supplemental campaigns vary in the age groups targeted but are assumed to have 100% efficacy, for example, in 2013 Afghanistan, reported MCV1 and MCV2 coverage of 68% and 54%, respectively. Therefore, our model would assume 0.85 × 68% of births that year and 0.95 × 54% of children aged 1 to 2 were vaccinated and immunized. The supplemental campaign that year targeted children between 9 months and 10 years of age and reported coverage of 93%. Thus, 93% of susceptible children between 9 months and 1 year were vaccinated and immunized. This was calculated as 93% of 25% of the 0 to 1 year age class. Additionally, 93% of the susceptible children between 1 year and 10 years was assumed to be vaccinated and immunized.

For all simulations, we assumed a population size of 1 million and a birth cohort each year of 50 000; this birthrate is consistent with Niger, Ethiopia, and Democratic Republic of the Congo, the three highest measles burden countries in sub‐Saharan Africa, in year 2000.^1^ We then simulated time series of cases for each of the 100 randomly selected combinations of parameters and vaccination programs or 33 years. For each simulation, we initialized the susceptible population age distribution as *N*_*a*_=0.25∗*N*_*a*−1_, where *N*_*a*_ is the population size in age class *a*; thus, this is consistent with a mean age of cases of 4 years, which is consistent with high‐burden measles settings.^15^ In Figure 3, the time series generated by these 100 combinations are pictured.

### 4.2 SIR model

The annual model presented earlier reflects an over simplification of the dynamics of measles transmission. The standard for simulating measles has been the SIR model framework, which represents dynamics on the time scale of the duration of infection in an individual, approximately 2 weeks. This modeling paradigm is overly complex and too highly resolved, for the annual reporting data in national measles surveillance. However, as the SIR framework has been shown to adequately reflect measles dynamics, we present a simulation study to illustrate the ability of our proposed annual model to reconstruct the incidence simulated from an SIR‐style model simulated at a finer than annual time scale.

We simulated measles dynamics using a discrete time age‐structured MSIRV model, the details of this model have been described elsewhere^16^ so we provide a short summary here with further details in the supplement. The probability of infection of a susceptible individual of age, *a*, is derived from population‐adjusted age‐specific contact rates. We assumed that the structure of the age‐specific contact patterns follows those characterized in the European POLYMOD study based on diary entries^17^ and scaled the probability of transmission, given contact, to achieve a specified level of *R*_0_ (see the work of Metcalf et al^16^ for details). We generated simulations assuming *R*_0_ between 16 and 20 to reflect realistic uncertainty in transmission.^7^ The model assumes 2‐week discrete time steps; thus, individuals progress directly from the susceptible to infectious class and leave the infectious class after 1 time step. While this formulation ignores the short exposed, but not infectious, period in measles infection, it has been previously shown to exhibit good fit to observed measles time series.^8^, ^18^, ^19^

Age classes are stratified into 225 age groups (monthly strata up to 15 years of age; yearly thereafter). In each age bin, the population is further divided into relevant epidemiological compartments (ie, maternally immune, susceptible, infected, recovered, and vaccinated). All individuals are assumed to be born with maternal immunity and its decay with age was modeled as a monthly exponential decline with a rate of 0.45, which translates into less than 5% maternally immune by 7 months.^12^ Following the decay of maternal immunity, individuals become susceptible, after which they can be vaccinated at an age‐specific rate, or infected at a rate that depends on the age‐specific transmission rate and the prevalence of infection (the fraction of the population that is infected) in each age class.

Routine vaccination is assumed to occur at an age‐specific hazard rate, with a maximum at 9 to 12 months of age; the relative age‐specific hazard of routine vaccination was extracted from empirical estimates for Zambian children from the work of Lessler et al.^20^ In each year of simulation, the hazard rate was scaled so that the proportion of children expected to be immunized by 24 months of age was equal to the coverage with the first dose of measles containing vaccine. The same was done for the second dose, with the change that the maximum rate was at 24 months and rate was scaled so that the proportion of children expected to be immunized by 36 months of age was equal to the coverage with the second dose of measles containing vaccine.

Our annual model is designed to be applied to national aggregate surveillance, which reflects the sum over many heterogeneous subnational units. The SIR model framework reflects a well‐mixed system^9^ and thus is unlikely to reflect the multiple sources of heterogeneity in a national aggregate. To account for this subnational variability, we simulated national aggregates as the sum of 10 time series, simulated independently using the MSIRV model. For each independent time series, we assumed total population of size 1 million and an annual birth cohort of 50 000 births, evenly distributed across each year. Thus, the national population size is 10 million. We drew routine first and second dose measles vaccination coverage at random from the time series of true countries (as earlier); thus, the national coverage for each dose in each year is the average over the 10 independent simulations. We assumed that SIA campaigns were synchronized over all 10 independent units.

We further simulated an additional 15 years of measles incidence, beyond year 33, for all 100 iterations assuming either an optimistic scenario in which MCV1 and MCV2 coverage are held constant at 93% and a pessimistic scenario in which MCV1 coverage is reduced to 75% and MCV2 is reduced to 0%. In both scenarios, we assume no additional SIAs and project case burden for 15 years. We then used the parameters estimated by fitting the annual model earlier, Equations 1, 2, and 3, to the first 33 years of simulations from the SIR model to project measles incidence for 15 years using the annual model.

## 5 RESULTS

**Figure 4:**
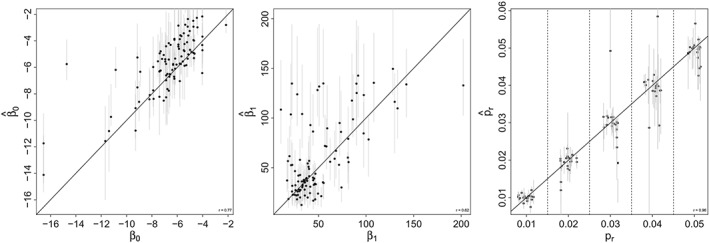
Estimates of parameter values from 100 simulated data sets from the annual model as a function of the true value used to simulate the data. A, The intercept of the attack rate function β_0_; B, The slope of the attack rate function β_1_; C, The reporting rate p_r_. The gray bars indicate the 95% CI based on the parametric bootstrap generated sampling distributions

**Figure 5:**
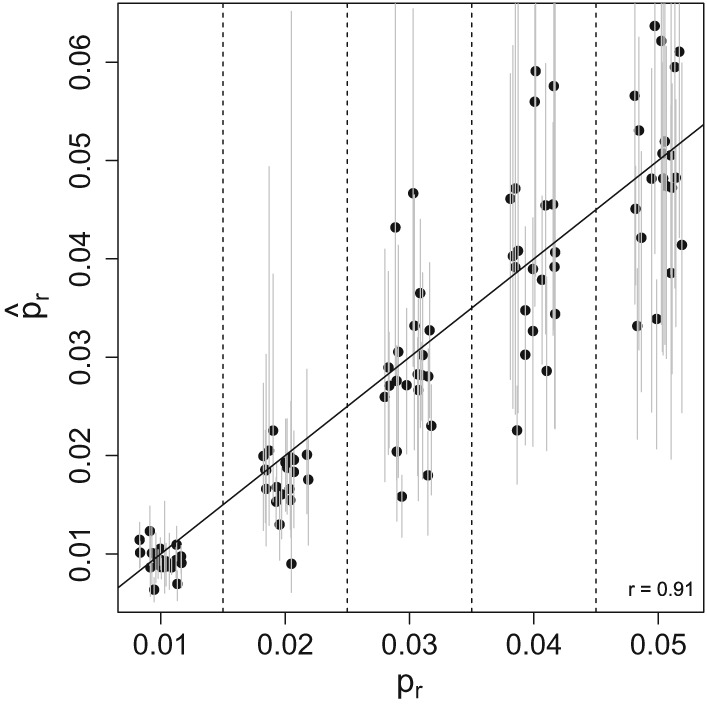
Estimates of the reporting rate, p_r_ from 100 simulated data sets from the Susceptible‐Infected‐Recovered model as a function of the true value used to simulate the data. The gray bars indicate the 95% CI based on the parametric bootstrap generated sampling distributions

The proposed fitting procedure when fitted to data simulated from the annual model gives parameter estimates that are positively correlated with the parameter values used in simulation (Figure 4). The correlation between estimates of *β*_0_ and *β*_1_ and the true values used in simulation was 0.77 and 0.62, respectively. There was a slight positive bias in the estimates of *β*_0_. The estimated reporting probability was strongly correlated with the true value used in simulation (correlation 0.96). Since the phenomenological attack rate model does not have a direct analog in the aggregated SIR simulations, it is not possible to directly assess the bias in estimates of *β*_0_ and *β*_1_. However, we are able to directly assess the estimates of the reporting probability. The correlation between the true reporting rate used in simulation and the estimate was 0.91 (Figure 5).

**Figure 6:**
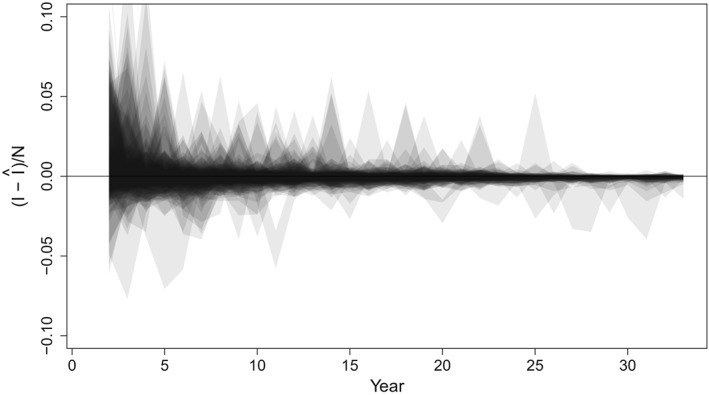
Normalized prediction intervals over 33 years of data from 100 simulations from the annual model simulated data sets. The y‐axis reflects the true incidence, minus the predicted incidence, as a fraction of population size to remove trends in individual time series. Shaded regions including the zero line indicate that the prediction interval captures the true value

**Figure 7:**
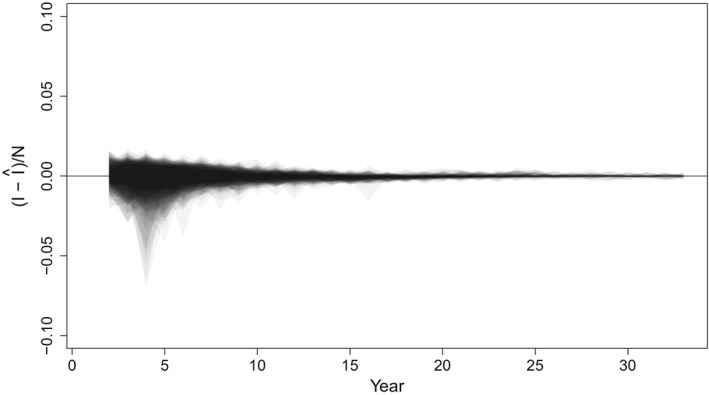
Normalized prediction intervals over 33 years of data from 100 simulations from the SIR model simulated data sets. The y‐axis reflects the true incidence, minus the predicted incidence, as a fraction of population size to remove trends in individual time series. Shaded regions including the zero line indicate that the prediction interval captures the true value

Because each simulated time series results in a different trajectory, we present prediction errors between the predicted and true number of measles cases, *I* (Figures 6 and 7). For both the annual and the SIR model simulations, the estimates of the true number of cases are centered at the unobserved truth. The width of confidence bounds tends to decrease over time, though this phenomenon can be attributed to the decreasing incidence of measles in all simulated scenarios; recall that vaccination coverage is drawn from observed country time series, which have tended to increase since 1980. For the annual model, the absolute error in estimates of the unobserved number of cases was within 0.1% of the population size (or 1000 cases) for 86.8% of the estimates over the full time series. Over the last 10 years of the time series, 98.7% of estimates were within 0.1% of the population size.

Predictions of the true unobserved states for simulations from the SIR model also tended to be close to the truth, with a slight negative bias, particularly late in the time series, when true numbers of cases are small. The absolute error in estimates of the unobserved number of cases was within 0.1% of the population size (or 52 000 cases) for 84.1% of the estimates over the full time series. Over the last 10 years of the time series, 88.1% of estimates were within 0.1% of the population size.

**Figure 8:**
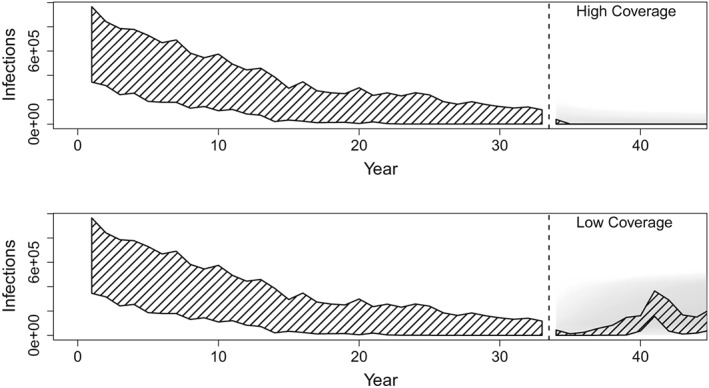
Infections, which are the total number of measles cases, are shown across years. Forward predictions of the unobserved number of infections from fitted model for the optimistic (top) and pessimistic (bottom) future vaccination scenarios. Hatched shading illustrates the central 95% of simulated incidence for 33 years of observed data (left of dashed line) and 15 years of future simulation (right of dashed line) from 100 simulations. Gray shading illustrates the central 95% of the prediction interval for 15 years of forward prediction for each of the 100 fitted models. Darker shading indicates higher density of predicted incidence

A natural application of this work is to perform short‐term forward projection of disease burden. A full exploration of the predictive ability of the fitted model for a range of future scenarios is beyond the scope of this analysis. However, we do illustrate a simple case study of model predictions for an optimistic (Figure 8A) and pessimistic scenario (Figure 8B). Hatched shading indicates the central 95% of simulated incidence for 100 iterations of the SIR metapopulation model; gray shading indicates the projected future incidence from the fitted model. Not surprisingly, when coverage is maintained at high levels, both simulation and predicted incidence remain low. In the pessimistic scenario, where vaccination coverage declines, the resulting predicted futures also increase. The absolute error in estimates of the unobserved number of cases was within 0.1% of the population size (or 52 000 cases) for 100% of the estimates over the full time series in the high coverage scenario. The absolute error in estimates of the unobserved number of cases was within 0.1% of the population size for 89.9% of the estimates over the full time series in the low coverage scenario. Predictions from the fitted model reflect the increase in mean incidence over a 7 to 10 year period, though clearly fail to capture the large fluctuation that occurred as a result of the resurgence of natural transmission over years 11 to 15.

## 6 DISCUSSION

Here, we have presented a model and accompanying statistical procedures for predicting the unobserved true incidence of measles infection from aggregated annual cases reported to the health system. Because reported cases are assumed to underrepresent the true number of cases occurring in the community, we employ a state‐space modeling framework that represents the reported cases as a binomial random variable drawn from the true unobserved number of cases at a reporting rate *p*_*r*_. The dynamics of measles persistence at the scale of national aggregates likely reflects the sum of many processes – seasonality in transmission, local fade‐out, and recolonization via metapopulation dynamics within the country^10^, ^21^ and importation from outside^13^ subnational heterogeneity in vaccination coverage.^22^ Furthermore, administrative aggregation of surveillance to annual sums is likely to eliminate, or at least dampen, the signal of many of these processes. Rather than attempt an explicit representation of these dynamics for all countries, we have presented a phenomenological model of the time series dynamics that includes indirect effects via a negative (positive) relationship between annual attack rate and the proportion of the population that is immune (susceptible). We illustrate, using simulated time series from both an annual model and a mechanistic SIR‐style metapopulation model, that the model and fitting method that we present produces approximately unbiased estimates of the reporting rate and the unobserved true number of measles cases.

Measles vaccination programs employ multiple doses of vaccine, either through a routine second dose or SIAs. Administrative estimates of coverage for these second dose opportunities do not explicitly account for the fraction of the population likely to be previously immunized through either vaccination or natural immunity.^6^ Here, we include a dynamic demographic model of the age distribution of susceptible individuals, which is depleted both through natural infection and prior vaccination. Thus, the resulting impact of second dose opportunities on the reduction of susceptible individuals is an emergent property of the dynamics themselves. At present, estimates of measles burden are based on non–age‐specific national aggregates of reported cases^4^; however, the incorporation of a demographic model for both the susceptible population and the resulting age distribution of cases, here, allows the possibility of formal model evaluation using age‐specific case reporting or serological surveys as these data become increasingly available.

The structural simplicity of the model we present here necessarily has limitations. In the model presented, we assume that reporting rate is constant over time. This is unlikely to be the case in practice as reporting rates may increase with health system development or at times of heightened awareness, such as outbreaks. Simons et al^1^ addressed this in their analysis and presented an alternative observation model (Equation 3) that assumed a higher reporting rate in years identified a priori, either because of outbreaks or other programmatic changes that resulted in high reporting rates. Incorporating such an alternative model in the analysis described earlier is straightforward. Furthermore, we assume, for simplicity, that the second dose opportunities for measles vaccination (via routine or SIAs) are independent of the first dose. However, it is possible that individuals who receive the first dose are more likely to also receive the second dose because of higher access to vaccination services, which would mean that coverage estimates for second dose opportunities are an overestimate of the resulting immunity.^6^, ^20^, ^23^ As with alternative assumptions about reporting rate, it is straightforward to alter the statement of Equation 1 to account for an assumption of non‐independent second dose opportunities.

These limitations speak to the utility of a simple phenomenological model. Addressing each of these points on a case‐by‐case basis for all countries is an intractable task, and any specific assumptions are likely to be violated in part. However, given the structural simplicity of the model presented, it is possible to represent various possible assumptions as alternative models within an ensemble that reflects, for example, alternative specifications of reporting rate (constant or increasing over time), the dependence of multiple doses, or measures of coverage (administrative estimates or estimates from coverage surveys^22^). The likelihood framework presented allows a formal quantification of the fit of each model within the ensemble which could permit ensemble estimates of burden, following the example of multimodel inference employed in medical diagnosis^24^ and climate science.^25^

A natural extension of these methods is to consider forward projection. We note that any forward projection is necessarily “out‐of‐sample” and should be interpreted with caution. A further full assessment of performance of forward projections is beyond the scope of this work. The prior method for measles burden estimation by Chen et al^5^ and Simons et al^1^ were poorly suited to forward projection as the underlying structure of the model (the EKF) was not naturally constrained, that is, the random component of both the process and observation models was Gaussian and could result in arbitrarily high or low (even negative) values without ad hoc corrections. The model we present here is based on a binomial distribution for both the process and the observation model, which is naturally constrained, eg, unobserved cases cannot be below 0 or greater than the number of susceptibles in the forward simulation step nor can they be less than the observed number of cases in the observation step. This requires that we use simulation‐based methods to estimate the likelihood (particle filter) rather than the analytic approximation of the likelihood used in the works of Chen et al^5^ and Simons et al^1^; however, it does provide natural demographic constraints on forward projections. We illustrate here that short term projections perform as expected: the mean number of cases scales with vaccination coverage and prediction bounds scale with incidence. We present this simple example of forward projection as a illustration of model consistency to show that the model fitted to data from the past generates intuitive projections when unconstrained by observed data. A full assessment of the performance of forward projections is the subject of ongoing work.

The estimation of global infectious disease burden is an important task, but necessarily hampered by idiosyncrasies of transmission dynamics and reporting, as well as data uncertainties at the local scale. Thus, any choice of model is unlikely to be perfectly applicable in all settings. Here, we present a simple framework for estimating the burden of measles disease that is optimized for estimating the unobserved incidence in the community from partially reported case data, rather than estimating fine‐scale mechanism. We illustrate the performance of the estimators on both an annual, phenomenological model, from which the estimators were derived, a fine‐scale mechanistic model that is consistent with the state‐of‐the‐art knowledge of measles transmission dynamics.

## CONFLICT OF INTEREST

We report no conflicts of interest.

## DATA AVAILABILITY STATEMENT

The data that support the findings of this study are available from the corresponding author upon reasonable request.

## References

[1] Simons E , Ferrari M , Fricks J , et al. Assessment of the 2010 global measles mortality reduction goal: results from a model of surveillance data. The Lancet. 2012;379(9832):2173‐2178. 10.1016/S0140-6736(12)60522-4 22534001

[2] Ozawa S , Clark S , Portnoy A , et al. Estimated economic impact of vaccinations in 73 low‐ and middle‐income countries, 2001‐2020. Bull World Health Organ. 2017;95(9):629‐638. 10.2471/BLT.16.178475 28867843 PMC5578376

[3] Grenfell BT , Anderson RM . The estimation of age‐related rates of infection from case notifications and serological data. Epidemiol Infect. 1985;95(2):419‐436. 10.1017/S0022172400062859 PMC2129533 4067297

[4] Dabbagh A , Patel MK , Dumolard L , et al. Progress toward regional measles elimination—worldwide, 2000‐2016. MMWR Morb Mortal Wkly Rep. 2017;66(42):1148‐1153. 10.15585/mmwr.mm6642a6 29073125 PMC5689104

[5] Chen S , Fricks J , Ferrari MJ . Tracking measles infection through non‐linear state space models. J R Stat Soc Ser C Appl Stat. 2012;61(1):117‐134. 10.1111/j.1467-9876.2011.01001.x

[6] Li S , Ma C , Hao L , et al. Demographic transition and the dynamics of measles in six provinces in China: a modeling study. PLOS MEDICINE. 2017;14(4). 10.1371/journal.pmed.1002255 PMC5380361 28376084

[7] Anderson R , May R . Infectious Diseases of Humans: Dynamics and Control. Oxford, UK: Oxford University Press; 1991.

[8] Finkenstädt BF , Grenfell BT . Time series modelling of childhood diseases: a dynamical systems approach. J R Stat Soc Ser C Appl Stat. 2000;49(2):187‐205. 10.1111/1467-9876.00187

[9] Begon M , Bennett M , Bowers RG , French NP , Hazel SM , Turner J . A clarification of transmission terms in host‐microparasite models: numbers, densities and areas. Epidemiol Infect. 2002;129(1):147‐153. 10.1017/S0950268802007148 12211582 PMC2869860

[10] Grenfell B , Harwood J . (Meta)population dynamics of infectious diseases. Trends Ecol Evol. 1997;12(10):395‐399. 10.1016/S0169-5347(97)01174-9 21238122

[11] Hanski I . Single‐species metapopulation dynamics: concepts, models and observations. Biol J Linn Soc. 1991;42(1‐2):17‐38. 10.1111/j.1095-8312.1991.tb00549.x

[12] Cáceres VM , Strebel PM , Sutter RW . Factors determining prevalence of maternal antibody to measles virus throughout infancy: a review. Clin Infect Dis. 2000;31(1):110‐119. 10.1086/313926 10913406

[13] Bednarczyk RA , Rebolledo PA , Omer SB . Assessment of the role of international travel and unauthorized immigration on measles importation to the United States. J Travel Med. 2016;23(3). 10.1093/jtm/taw019 27029908

[14] Doucet A , de Freitas N , Gordon N . Sequential Monte Carlo Methods in Practice. Berlin, Germany: Springer; 2001.

[15] Ferrari MJ , Grenfell BT , Strebel PM . Think globally, act locally: the role of local demographics and vaccination coverage in the dynamic response of measles infection to control. Philos Trans R Soc B Biol Sci. 2013;368(1623). 10.1098/rstb.2012.0141 PMC3720039 23798689

[16] Metcalf CJE , Lessler J , Klepac P , Morice A , Grenfell BT , Bjørnstad ON . Structured models of infectious disease: inference with discrete data. Theor Popul Biol. 2012;82(4):275‐282. 10.1016/j.tpb.2011.12.001 22178687 PMC4086157

[17] Mossong J , Hens N , Jit M , et al. Social contacts and mixing patterns relevant to the spread of infectious diseases. PLOS MEDICINE. 2008;5(3). 10.1371/journal.pmed.0050074 PMC2270306 18366252

[18] Ferrari MJ , Grais RF , Bharti N , et al. The dynamics of measles in sub‐Saharan Africa. Nature. 2008;451(7179):679‐684. 18256664 10.1038/nature06509

[19] Metcalf CJE , Bjørnstad ON , Grenfell BT , Andreasen V . Seasonality and comparative dynamics of six childhood infections in pre‐vaccination Copenhagen. Proc R Soc B Biol Sci. 2009;276(1676):4111‐4118. 10.1098/rspb.2009.1058 PMC2821338 19740885

[20] Lessler J , Metcalf CJE , Grais RF , Luquero FJ , Cummings DA , Grenfell BT . Measuring the performance of vaccination programs using cross‐sectional surveys: a likelihood framework and retrospective analysis. PLOS MEDICINE. 2011;8(10). 10.1371/journal.pmed.1001110 PMC3201935 22039353

[21] Gunning CE , Ferrari MJ , Erhardt EB , Wearing HJ . Evidence of cryptic incidence in childhood diseases. Proc R Soc B Biol Sci. 2017;284(1861). 10.1098/rspb.2017.1268 PMC5577489 28855364

[22] Takahashi S , Metcalf CJE , Ferrari MJ , Tatem AJ , Lessler J . The geography of measles vaccination in the African Great Lakes region. Nature Communications. 2017;8 10.1038/ncomms15585 PMC5458501 28541287

[23] McKee A , Shea K , Ferrari MJ . Optimal vaccine schedules to maintain measles elimination with a two‐dose routine policy. Epidemiol Infect. 2017;145(2):227‐235. 10.1017/S0950268816002296 27760574 PMC5197928

[24] West D , Mangiameli P , Rampal R , West V . Ensemble strategies for a medical diagnostic decision support system: a breast cancer diagnosis application. Eur J Oper Res. 2005;162(2):532‐551. 10.1016/j.ejor.2003.10.013

[25] Chandler RE . Exploiting strength, discounting weakness: combining information from multiple climate simulators. Philos Trans R Soc A Math Phys Eng Sci. 2013;371(1991). 10.1098/rsta.2012.0388 23588053

